# Evaluation of Oxidative Stress and Its Impact on Sperm DNA Fragmentation and Semen Parameters in Infertile Men

**DOI:** 10.1155/tswj/8887567

**Published:** 2026-08-24

**Authors:** Kenza Berrada, Yasmine Touhamia, Asmaa Serbouti, Abderrahmane Aamiri, Fatima Ez-Zahra Ousaid, Samy Housbane, Mostafa Kabine, Noureddine Louanjli, Rachid Aboutaieb

**Affiliations:** ^1^ Laboratory of Sexual and Reproductive Health, Faculty of Medicine and Pharmacy, Hassan II University, Casablanca, Morocco, univh2c.ma; ^2^ Health, Environment and Biotechnology Laboratory, Faculty of Sciences Ain Chock, Hassan II University, Casablanca, Morocco, uh2c.ac.ma; ^3^ Department of Medical Informatics, Faculty of Medicine and Pharmacy, Hassan II University, Casablanca, Morocco, univh2c.ma; ^4^ Labomac Laboratory of Medical Analysis and Reproductive Biology, Casablanca, Morocco; ^5^ Department of Urology, Ibn Rochd University Hospital Center, Casablanca, Morocco, chuibnrochd.ma

**Keywords:** antioxidant, infertile men, malondialdehyde, oxidative stress, serum testosterone, sperm DNA fragmentation

## Abstract

Oxidative stress, defined as an imbalance between reactive oxygen species (ROS) production and antioxidant defenses, plays a central role in the pathophysiology of male infertility and has been reported in approximately 30%–80% of infertile men. Excessive ROS production promotes lipid peroxidation, DNA damage, and mitochondrial dysfunction, ultimately compromising sperm quality. The aim of our study was to evaluate oxidative stress biomarkers in seminal plasma, including malondialdehyde (MDA) as a marker of lipid peroxidation and key enzymatic antioxidants superoxide dismutase (SOD), catalase (CAT), and glutathione peroxidase (GPx), and to assess their impact on sperm DNA fragmentation, semen parameters, and serum total testosterone levels. Semen samples were collected from a total of 150 participants, including 120 infertile men (30 oligozoospermic, 30 teratozoospermic, 30 asthenozoospermic, and 30 necrozoospermic) and 30 fertile controls. Semen analysis was performed according to WHO 2021 criteria. Sperm DNA fragmentation index (DFI) was assessed using the TUNEL assay. Seminal antioxidant enzyme activities (SOD, CAT, and GPx) and lipid peroxidation levels were measured in seminal plasma using spectrophotometric methods. Serum total testosterone levels were measured using an electrochemiluminescence immunoassay. The results showed that DFI and MDA levels were significantly higher in infertile men compared with controls. Conversely, seminal antioxidant enzyme activities and serum testosterone levels were significantly lower in the infertile groups (*p* < 0.05). DFI was positively correlated with MDA (*r* = 0.63, *p* < 0.001). In contrast, reduced seminal antioxidant enzyme activities and lower testosterone levels were negatively associated with higher DFI and MDA (*p* < 0.001). Additionally, both DFI and MDA showed negative correlations with semen parameters. Our findings highlight the potential value of oxidative stress biomarkers and sperm DNA fragmentation assessment in the evaluation of male infertility, emphasizing the central role of oxidative stress in sperm dysfunction.

## 1. Introduction

Infertility is a major clinical problem and a global public health issue. The World Health Organization (WHO) defines infertility as the inability to achieve pregnancy after 12 months of regular, unprotected sexual intercourse [1]. It is estimated to affect approximately 15% of couples of reproductive age, with male factors accounting for nearly 50% of cases [2]. Among the various mechanisms implicated, oxidative stress (OS) is particularly important, as it has been reported in 30%–80% of infertile men [3].

OS results from an imbalance between excessive production of reactive oxygen species (ROS) and insufficient antioxidant defenses [4, 5]. Spermatozoa are especially vulnerable to OS because their plasma membrane is rich in polyunsaturated fatty acids (PUFAs) and they possess a limited capacity for DNA repair, which makes them highly susceptible to oxidation [6].

Increased ROS levels exacerbate this vulnerability by targeting several critical structures of the sperm cell [7]. At the plasma membrane level, OS induces lipid peroxidation by attacking PUFAs, leading to membrane destabilization and reduced sperm–oocyte fusion capacity [8]. OS also induces sperm DNA fragmentation, resulting in both single‐ and double‐strand breaks, as well as oxidative modifications of nucleotide bases. This molecular damage may further contribute to telomere shortening and epigenetic alterations [9]. At the sperm midpiece, OS impairs mitochondrial function, leading to reduced ATP production and compromised flagellar motility [10]. Furthermore, OS disrupts testicular steroidogenesis by impairing Leydig cell function, resulting in decreased testosterone levels [11]. Collectively, these alterations contribute to a decline in sperm quality, including motility, viability, and morphology, thereby reducing fertilizing potential and contributing to the failure of assisted reproductive techniques [12].

To protect spermatozoa from ROS‐induced damage, seminal plasma is equipped with a wide range of antioxidant systems. These include enzymatic antioxidants such as superoxide dismutase (SOD), catalase (CAT), and glutathione peroxidase (GPx). SOD constitutes the first line of antioxidant defense by converting superoxide radicals into hydrogen peroxide (H_2_O_2_). CAT subsequently decomposes H_2_O_2_ into water and oxygen, whereas GPx reduces H_2_O_2_ and lipid hydroperoxides, thereby protecting spermatozoa from oxidative damage. The activity of these enzymes depends on several cofactors, including selenium, zinc, and copper, which contribute to maintaining cellular redox homeostasis [13]. Nonenzymatic antioxidants also play a crucial role, comprising endogenous molecules like glutathione and coenzyme Q10, as well as dietary components such as vitamins A, C and E, carotenoids, and polyphenols [14]. The synergistic action of these systems helps maintain redox balance and preserve the structural and functional integrity of spermatozoa [15].

Considering the critical role of OS and sperm DNA fragmentation as key markers of male infertility, and the protective function of seminal antioxidants, this study is aimed at quantifying enzymatic antioxidants (SOD, GPx, and CAT) and lipid peroxidation (MDA) in seminal plasma, and to investigate their correlations with sperm DNA fragmentation, semen quality, and serum testosterone levels, thereby providing insights into the oxidative mechanisms underlying male infertility.

## 2. Materials and Methods

### 2.1. Study Design

A total of 150 volunteers (aged 27–45 years) were included in a prospective observational cross‐sectional study conducted at the Laboratory of Medical Analysis and Reproductive Biology, Labomac, Casablanca, Morocco. Participants were recruited from a Moroccan population. To minimize potential confounding factors, individuals with known lifestyle risk factors such as smoking and alcohol consumption were excluded from the study.

This study was conducted between April 2022 and December 2024, in collaboration with the Laboratory of Health, Environment and Biotechnology, Faculty of Sciences Ain Chock, Hassan II University of Casablanca, Morocco. Participants were classified into five groups according to the WHO 6th edition 2021 semen analysis criteria: the oligozoospermic group (*n* = 30; sperm concentration < 16 million/mL), the asthenozoospermic group (*n* = 30; progressive motility [PR] < 30%), the teratozoospermic group (*n* = 30; normal morphology < 4%), and the necrozoospermic group (*n* = 30; vitality < 54%). Additionally, 30 fertile men with proven fertility and normozoospermic semen profiles were included as a control group, in order to assess differences between fertile and infertile men. The control group consisted of fertile men with recently proven fertility who had fathered a child within the year prior to enrollment and served as the reference group.

### 2.2. Exclusion Criteria

Exclusion criteria included subjects with azoospermia; those taking medications or antioxidant supplements during the study period; individuals with a history of infertility; patients with chronic diseases (such as diabetes, hypotension or hypertension, or undergoing hemodialysis); those exposed to gonadotoxins (chemotherapy, radiotherapy, or pesticides); individuals with leukocytospermia (>1 × 10^6^/mL); those with testicular varicocele or genital infections; as well as men who were smokers, alcohol consumers, or obese. In addition, individuals with occupational exposure to toxic agents, as well as those with known endocrine or thyroid disorders, were excluded from the study.

### 2.3. Ethics

The study was approved by the Ethics Committee of the Ibn Rochd University Hospital Center in Casablanca (Ref: 2022/60.CEH.S.KS1.59), according to the committee′s report dated April 22, 2022 (File No. 12/2022). The study was conducted in accordance with the ethical standards of the institutional and national research committees and with the Declaration of Helsinki (1975), as revised in 2013. Written informed consent was obtained from all participants prior to their inclusion in the study.

### 2.4. Sample Collection and Semen Analysis

Semen analyses were performed according to the WHO 6th edition 2021 guidelines [16]. Semen samples were obtained after 3–5 days of sexual abstinence and collected into sterile, properly labeled containers. For the liquefaction step, samples were incubated at 37°C for 60 min, and liquefaction was checked every 10 min until completion. The analysis of seminal parameters, including volume, concentration, motility, vitality, and morphology, was performed using a computer‐assisted semen analyzer (CASA; Microptic, Barcelona, Spain) [17]. Sperm motility was classified into three categories: PR, nonprogressive motility (NP), and immotile spermatozoa (IM). Sperm vitality was assessed using 2% eosin staining. A semen aliquot was mixed with eosin, and at least 200 spermatozoa were examined under a light microscope. Viable spermatozoa remained unstained, whereas nonviable spermatozoa appeared red. Sperm vitality was expressed as the percentage of unstained spermatozoa. Sperm morphology was evaluated on 2 × 200 spermatozoa per sample. Results were expressed as the percentage of abnormal morphology (complementary to the percentage of normal morphology). Smears were fixed and stained using the Shorr‐hematoxylin technique, allowing classification of morphological abnormalities according to their location (head, midpiece, and flagellum). After the assessment of sperm DNA integrity, raw semen samples were centrifuged at 1000 g for 10 min. The resulting seminal plasma was stored at −20°C for subsequent analysis of OS biomarkers. All analyses were performed by the same operator, and samples were analyzed in duplicate.

### 2.5. Assessment of Leukocytospermia

Leukocytospermia was defined as ≥1 × 10^6^ leukocytes/mL according to WHO 2021 criteria [16] and evaluated using the LeucoScreen test (FertiPro NV, Beernem, Belgium). Briefly, 10 *μ*L of semen was mixed with 10 *μ*L of working solution, incubated for 2 min, and examined under a light microscope (×400). Peroxidase‐positive leukocytes were identified by their brown coloration, counted manually, and expressed as cells/mL.

### 2.6. DNA Fragmentation Index (DFI) by Terminal Deoxynucleotidyl Transferase dUTP Nick‐End Labeling (TUNEL) Assay

Sperm DNA integrity was assessed using the TUNEL assay with the In Situ Cell Death Detection Kit, fluorescein (Roche Diagnostics GmbH, Mannheim, Germany). This method is based on the activity of terminal deoxynucleotidyl transferase (TdT), which catalyzes the incorporation of fluorescein‐labeled dUTP into the free 3 ^′^‐OH ends of fragmented DNA, allowing the detection of sperm DNA fragmentation [18]. Semen samples were diluted in phosphate‐buffered saline (PBS) and centrifuged for 10 min. After removal of the supernatant, the cell pellets were smeared onto slides, air‐dried, and fixed in formaldehyde prepared in PBS for 30 min. The slides were then rinsed and permeabilized with a solution containing Triton X‐100 and sodium citrate. After incubation for 1 min at room temperature, the slides were air‐dried. In the dark, the fluorescein‐labeled TUNEL reaction mixture was applied to the smears. The slides were covered with coverslips and incubated at 37°C for 45 min. After incubation, the slides were rinsed, mounted with glycerol, and examined under a fluorescence microscope. Spermatozoa exhibiting green fluorescence were considered to have fragmented DNA, whereas nonfluorescent spermatozoa were considered to have intact DNA. For each sample, at least 500 spermatozoa were evaluated [19]. The sperm DFI was calculated as follows:

DFI %=number of fluorescent spermatozoaTotal number of spermatozoa counted×100.

A DFI ≥ 30% was considered abnormal and indicative of impaired sperm nuclear integrity.

### 2.7. Serum Total Testosterone Assay

Morning fasting venous blood samples were collected between 08:00 and 10:30 from all participants. Serum total testosterone levels were measured using an electrochemiluminescence immunoassay (ECLIA) on the cobas e analyzer (Roche Diagnostics, Mannheim, Germany), according to the manufacturer′s instructions. This competitive immunoassay is based on the binding of testosterone in the sample to a ruthenium‐labeled monoclonal antibody, followed by electrochemiluminescence detection. Results were expressed in ng/mL.

### 2.8. Malondialdehyde Assay

Lipid peroxidation was assessed by measuring thiobarbituric acid reactive substances (TBARS) according to the method described by Samokyszyn and Marnett [20]. After liquefaction, semen samples were centrifuged at 1000 × g for 10 min to obtain seminal plasma. Subsequently, 100 *μ*L of seminal plasma was mixed with a solution containing 0.67% thiobarbituric acid (TBA) and 10% trichloroacetic acid (TCA) prepared in 0.25 M hydrochloric acid (HCl). The mixture was incubated in a boiling water bath (100°C) for 15 min, cooled in an ice bath, and centrifuged again at 1000 × g for 10 min. The absorbance of the supernatant was measured at 535 nm, and the results were expressed as nmol/mL.

### 2.9. CAT Activity

CAT activity was measured according to the method described by Aebi [21]. The reaction mixture consisted of 50 mM phosphate buffer (pH 7.0) and 50 *μ*L of seminal plasma. The reaction was initiated by the addition of 7.5 mM H_2_O_2_, and CAT activity was determined by monitoring the decrease in absorbance at 240 nm, corresponding to the decomposition of H_2_O_2_. Specific activity was expressed as U/mg protein.

### 2.10. SOD Activity

SOD activity was measured according to the method described by Paoletti and Mocali [22]. The reaction mixture contained 5 mM EDTA, 2.5 mM MnCl_2_, 0.27 mM NADH, 3.9 mM 2‐mercaptoethanol, 50 mM phosphate buffer (pH 7.0), and 50 *μ*L of seminal plasma. Enzyme activity was monitored spectrophotometrically at 340 nm, and the results were expressed as U/mg protein.

### 2.11. Gpx Activity

GPx activity was determined according to the method described by Flohé and Günzler [23]. The reaction mixture consisted of 50 mM phosphate buffer (pH 7.0), 1 mM EDTA, 0.45 mM reduced glutathione (GSH), 0.20 mM NADPH, 1.5 mM sodium azide, 0.45 U glutathione reductase, and 30 *μ*L of seminal plasma. The reaction was initiated by adding 0.72 mM H_2_O_2_. GPx activity was determined by monitoring the decrease in absorbance at 340 nm, corresponding to the oxidation of NADPH to NADP^+^ in the presence of glutathione reductase. Specific activity was expressed as U/mg protein.

### 2.12. Protein Assay

Protein concentration was determined according to the method described by Bradford [24], using bovine serum albumin (BSA) as the standard. Protein concentrations were used to normalize the activities of SOD, CAT, and GPx, which were expressed as U/mg protein.

### 2.13. Statistical Analysis

Statistical analyses were performed using RStudio software (Version 4.4.2). Data normality was assessed for each variable using the Shapiro–Wilk test. For normally distributed data (*p* ≥ 0.05), one‐way analysis of variance (ANOVA) followed by Tukey′s honestly significant difference (HSD) post hoc test was applied. When the assumption of normality was not met (*p* < 0.05), the Kruskal–Wallis test was used, followed by pairwise comparisons with Holm adjustment. Correlation analyses were performed on the pooled dataset including all participants. For each pair of variables, normality was first assessed using the Shapiro–Wilk test. Pearson′s correlation coefficient was applied when both variables were normally distributed, whereas Spearman′s rank correlation coefficient was used when at least one variable did not follow a normal distribution. Principal component analysis (PCA) was conducted on the standardized dataset using the prcomp function, and results were visualized through biplots generated with the factoextra and ggplot2 packages. Between‐group comparisons were illustrated using boxplots generated with the ggplot2 package. Statistical significance was set at *p* < 0.05.

## 3. Results

### 3.1. Data Summary of Participants According to Semen Parameters and Serum Testosterone Levels

**Figure 1:**
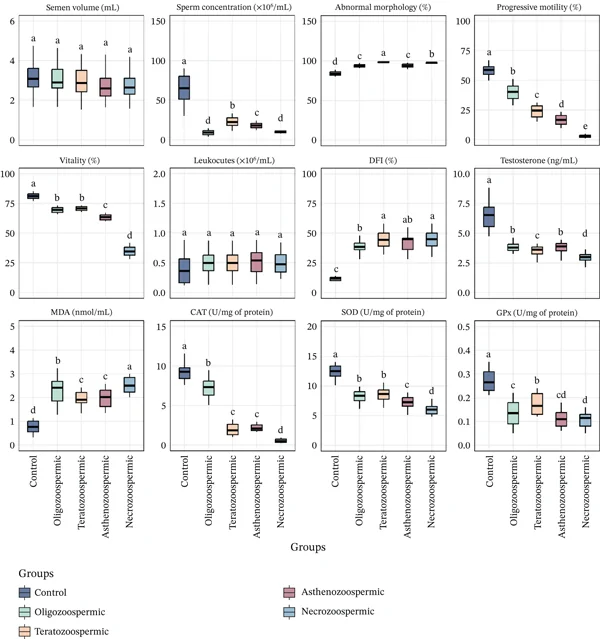
Seminal parameters, MDA (malondialdehyde), CAT (catalase), SOD (superoxide dismutase), GPx (glutathione peroxidase), sperm DNA fragmentation index (DFI, %), and serum testosterone levels in fertile controls and infertile groups. Letter labels (a–e) indicate statistically significant differences at *p* < 0.05 according to Tukey′s HSD post hoc test; groups sharing the same letter are not significantly different. Data are presented as median with interquartile range.

Statistical descriptions of age, semen parameters, and serum testosterone levels for the infertile groups and fertile controls are presented in Table 1 and Figure 1. This study included 150 volunteers. The age of the participants ranged from 35.70 ± 5.27 to 38.43 ± 4.17 years, with no significant differences between the fertile and infertile groups (*p* > 0.05). The period of sexual abstinence before semen collection was comparable among all groups, averaging approximately 4 days (3.57 ± 0.82 to 3.90 ± 0.88 days). Ejaculate volume also remained similar across the study groups, ranging from 2.72 ± 0.68 mL to 3.16 ± 0.70 mL. Leukocyte concentrations were below 1 × 10^6^/mL in all groups and did not differ significantly (*p* > 0.05), ranging from 0.40 to 0.52 × 10^6^/mL, indicating the absence of leukocytospermia and suggesting the absence of a significant inflammatory response.

**Table 1:** Seminal characteristics and serum testosterone levels between control and infertile groups.

Parameters	Control (*n* = 30)	Oligozoospermic (*n* = 30)	Teratozoospermic (*n* = 30)	Asthenozoospermic (*n* = 30)	Necrozoospermic (*n* = 30)
Age (years)	35.70 ± 5.27^a^	37.73 ± 5.69^a^	36.80 ± 4.62^a^	38.43 ± 4.17^a^	38.00 ± 4.85^a^
Abstinence (days)	3.90 ± 0.88^a^	3.63 ± 0.81^a^	3.57 ± 0.82^a^	3.63 ± 0.85^a^	3.70 ± 0.88^a^
Semen volume (mL)	3.16 ± 0.70^a^	3.07 ± 0.78^a^	2.94 ± 0.80^a^	2.74 ± 0.73^a^	2.72 ± 0.68^a^
Sperm concentration (×10^6^/mL)	65.06 ± 18.27^a^	9.12 ± 3.12^d^	22.40 ± 6.17^b^	18.07 ± 3.42^c^	10.45 ± 2.34^d^
Abnormal morphology (%)	84.02 ± 2.63^d^	93.80 ± 1.85^c^	98.43 ± 0.69^a^	93.77 ± 2.50^c^	97.54 ± 0.52^b^
Progressive motility (PR, %)	58.23 ± 4.69^a^	39.95 ± 6.39^b^	23.72 ± 5.13^c^	16.75 ± 4.29^d^	2.83 ± 1.13^e^
Vitality (%)	81.05 ± 2.34^a^	69.42 ± 2.36^b^	70.64 ± 1.80^b^	63.40 ± 2.28^c^	34.82 ± 4.13^d^
Leukocytes (×10^6^/mL)	0.40 ± 0.26^a^	0.51 ± 0.20^a^	0.51 ± 0.20^a^	0.52 ± 0.21^a^	0.50 ± 0.19^a^
Testosterone (ng/mL)	6.44 ± 0.96^a^	3.73 ± 0.61^b^	3.41 ± 0.61^c^	3.71 ± 0.58^b^	3.01 ± 0.43^d^

*Note:* Letter labels (a–e) on bars indicate statistical significance based on Tukey′s HSD post hoc test (*p* < 0.05). Groups sharing the same letter are not significantly different from each other. Values are presented as mean ± standard deviation.

Compared with the fertile control group, all infertile groups exhibited significantly lower sperm concentration, progressive motility, vitality, and serum total testosterone levels (*p* < 0.001). Conversely, abnormal sperm morphology was significantly higher in infertile men, with the highest values observed in the teratozoospermic group (*p* < 0.001). The necrozoospermic group showed the most severe impairment in sperm concentration, progressive motility, and vitality (Table 1). Oligozoospermic men were mainly characterized by a marked reduction in sperm concentration, whereas asthenozoospermic participants exhibited the greatest impairment in progressive motility.

### 3.2. OS Biomarkers and Sperm DNA Fragmentation

Table 2 and Figure 1 summarize the statistical comparisons of seminal antioxidant status, MDA concentrations, and sperm DFI among the fertile control and infertile groups. Lipid peroxidation, assessed by MDA levels, was significantly increased in all infertile groups compared with the fertile control group (0.75 ± 0.27 nmol/mL; *p* < 0.05). The highest MDA concentration was observed in the necrozoospermic group (2.51 ± 0.33 nmol/mL). Conversely, the activities of the antioxidant enzymes CAT, SOD, and GPx were significantly reduced in all infertile groups compared with the fertile control group (*p* < 0.001). Similarly, the sperm DFI was significantly increased in all infertile groups compared with the fertile control group (*p* < 0.001). Notably, DFI values exceeded the clinical threshold of 30% in all infertile groups, indicating severe impairment of sperm DNA integrity. The highest DFI values were observed in the necrozoospermic and teratozoospermic groups, followed by the asthenozoospermic and oligozoospermic groups.

**Table 2:** Lipid peroxidation, antioxidant enzyme activities, and sperm DNA fragmentation in fertile controls and infertile groups.

Biomarkers	Control (*n* = 30)	Oligozoospermic (*n* = 30)	Teratozoospermic (*n* = 30)	Asthenozoospermic (*n* = 30)	Necrozoospermic (*n* = 30)
MDA (nmol/mL)	0.75 ± 0.27^d^	2.30 ± 0.51^b^	1.97 ± 0.30^c^	1.98 ± 0.39^c^	2.51 ± 0.33^a^
CAT (U/mg protein)	9.24 ± 0.94^a^	7.20 ± 1.20^b^	1.94 ± 0.70^c^	2.20 ± 0.43^c^	0.55 ± 0.23^d^
SOD (U/mg protein)	12.40 ± 1.18^a^	8.21 ± 1.00^b^	8.54 ± 1.07^b^	7.22 ± 0.99^c^	5.92 ± 0.78^d^
GPx (U/mg protein)	0.27 ± 0.04^a^	0.13 ± 0.05^c^	0.17 ± 0.05^b^	0.11 ± 0.04^cd^	0.11 ± 0.04^d^
DFI (%)	11.50 ± 1.31^c^	38.73 ± 4.82^b^	44.17 ± 6.97^a^	42.20 ± 7.40^ab^	44.43 ± 6.46^a^

*Note:* Letter labels (a–e) indicate statistically significant differences at *p* < 0.05 according to Tukey′s HSD post hoc test. Groups sharing the same letter are not significantly different. Values are presented as mean ± standard deviation.

Abbreviations: CAT, catalase; DFI (%), DNA fragmentation index; GPx, glutathione peroxidase; MDA, malondialdehyde; SOD, superoxide dismutase.

### 3.3. Correlations Between OS Biomarkers, Semen Parameters, Sperm DNA Fragmentation, and Serum Total Testosterone Levels

Correlation coefficients between OS biomarkers, semen parameters, sperm DNA fragmentation, and serum total testosterone levels are presented in Table 3. Correlation analyses were performed on 150 semen samples. The strength of the associations was evaluated using the correlation coefficient (r) and the corresponding *p* value.

**Table 3:** Correlations between oxidative stress biomarkers, sperm parameters, DNA fragmentation, and serum total Testosterone levels.

Variables	MDA	CAT	SOD	GPx	Concentration	Motility	Vitality	Abnormal morphology	DFI	Testosterone
MDA	—	–0.44 ^∗∗^	–0.38 ^∗^	–0.44 ^∗∗^	–0.46 ^∗∗^	–0.45 ^∗∗^	–0.44 ^∗∗^	0.46 ^∗∗^	0.63 ^∗∗∗^	–0.46 ^∗∗^
CAT	–0.44 ^∗∗^	—	0.74 ^∗∗∗^	0.59 ^∗∗^	0.59 ^∗∗^	0.92 ^∗∗∗^	0.71 ^∗∗∗^	–0.60 ^∗∗^	–0.55 ^∗∗^	0.69 ^∗∗∗^
SOD	–0.38 ^∗^	0.74 ^∗∗∗^	—	0.72 ^∗∗∗^	0.76 ^∗∗∗^	0.83 ^∗∗∗^	0.75 ^∗∗∗^	–0.73 ^∗∗∗^	–0.50 ^∗∗^	0.72 ^∗∗∗^
GPx	–0.44 ^∗∗^	0.59 ^∗∗^	0.72 ^∗∗∗^	—	0.70 ^∗∗∗^	0.68 ^∗∗∗^	0.58 ^∗∗^	–0.64 ^∗∗^	–0.51 ^∗∗^	0.64 ^∗∗^
Concentration	–0.46 ^∗∗^	0.59 ^∗∗^	0.76 ^∗∗∗^	0.70 ^∗∗∗^	—	0.68 ^∗∗∗^	0.57 ^∗∗^	–0.74 ^∗∗∗^	–0.63 ^∗∗∗^	0.78 ^∗∗∗^
Progressive Motility	–0.45 ^∗∗^	0.92 ^∗∗∗^	0.83 ^∗∗∗^	0.68 ^∗∗∗^	0.68 ^∗∗∗^	—	0.83 ^∗∗∗^	–0.68 ^∗∗∗^	–0.57 ^∗∗^	0.74 ^∗∗∗^
Vitality	–0.44 ^∗∗^	0.71 ^∗∗∗^	0.75 ^∗∗∗^	0.58 ^∗∗^	0.57 ^∗∗^	0.83 ^∗∗∗^	—	–0.61 ^∗∗^	–0.42 ^∗^	0.58 ^∗∗^
Abnormal morphology	0.46 ^∗∗^	–0.60 ^∗∗^	–0.73 ^∗∗∗^	–0.64 ^∗∗^	–0.74 ^∗∗∗^	–0.68 ^∗∗∗^	–0.61 ^∗∗^	—	0.64 ^∗∗∗^	–0.64 ^∗∗^
DFI	0.63 ^∗∗∗^	–0.55 ^∗∗^	–0.50 ^∗∗^	–0.51 ^∗∗^	–0.63 ^∗∗∗^	–0.57 ^∗∗^	–0.42 ^∗^	0.64 ^∗∗∗^	—	–0.62 ^∗∗^
Testosterone	–0.46 ^∗∗^	0.69 ^∗∗∗^	0.72 ^∗∗∗^	0.64 ^∗∗^	0.78 ^∗∗∗^	0.74 ^∗∗∗^	0.58 ^∗∗^	–0.64 ^∗∗^	–0.62 ^∗∗^	—

*Note:*Significant correlations: ∗ *p* < 0.05; ∗∗ *p* <0.001; ∗∗∗ *p* <0.0001.

Abbreviations: CAT, catalase; DFI, DNA fragmentation index; GPx, glutathione peroxidase; MDA, malondialdehyde; SOD, superoxide dismutase.

MDA showed a strong positive correlation with DFI (*r* = 0.63, *p* < 0.0001), indicating that higher OS is accompanied by reduced DNA integrity and increased sperm DNA fragmentation. Furthermore, DFI was significantly negatively correlated with sperm concentration (*r* = –0.63, *p* < 0.0001), progressive motility (*r* = –0.57, *p* < 0.001), and vitality (*r* = –0.42, *p* < 0.05), whereas a positive correlation was observed with abnormal morphology (*r* = 0.64, *p* < 0.0001). These findings suggest that sperm DNA damage is closely associated with impaired semen quality and structural sperm abnormalities. In addition, MDA demonstrated significant negative correlations with sperm concentration (r = –0.45, *p* < 0.001), progressive motility (r = –0.45, *p* < 0.001), and vitality (*r* = –0.44, *p* < 0.001), whereas it was positively associated with abnormal morphology (*r* = 0.45, *p* < 0.001). These findings indicate that increased OS is associated with reduced sperm concentration, impaired motility, decreased vitality, and poorer sperm morphology. Moreover, MDA was negatively correlated with serum testosterone levels (*r* = –0.46, *p* < 0.001), suggesting that increased OS is associated with reduced serum total testosterone levels. Antioxidant enzymes (SOD, CAT, and GPx) showed similar positive correlations with semen parameters and serum total testosterone levels, indicating that higher antioxidant activity is associated with improved semen quality and higher serum total testosterone levels. All antioxidant enzymes were inversely correlated with MDA levels, indicating an imbalance between antioxidant defenses and OS. Additionally, SOD, CAT, and GPx were negatively correlated with DFI (*r* = –0.55, –0.50, and –0.51, respectively; *p* < 0.001), highlighting their protective effects against sperm nuclear damage. Finally, all three antioxidant enzymes were positively correlated with serum total testosterone levels (*p* < 0.001), suggesting a close association between antioxidant defense and hormonal status.

**Figure 2:**
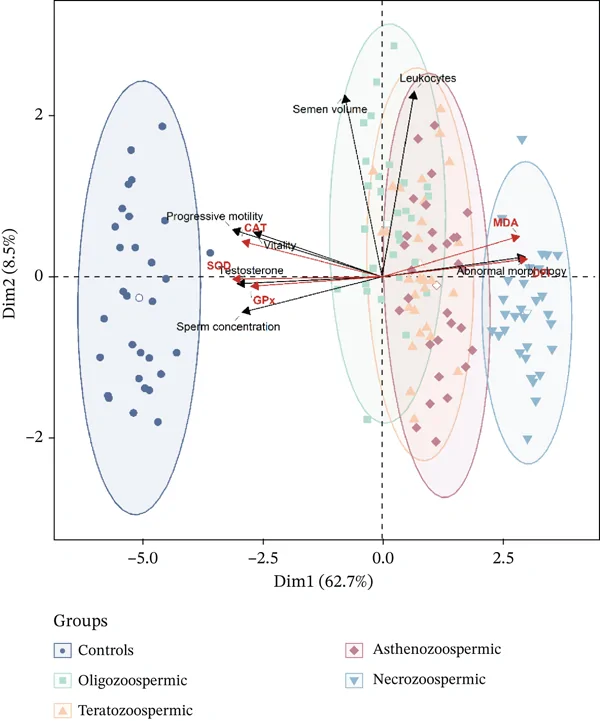
Principal component analysis (PCA) biplot illustrating the distribution of semen samples and the contribution of seminal parameters, oxidative stress biomarkers, sperm DNA fragmentation index (DFI), and hormonal levels across the study groups.

The PCA biplot (Figure 2) displays the projection of semen samples (individuals) and variables (semen parameters, OS biomarkers, and serum total testosterone levels) onto the first two principal components. Dim1 explained 62.7% of the total variance and primarily separated the fertile control group from the infertile groups, whereas Dim2 explained 8.5% of the total variance. Progressive motility, vitality, sperm concentration, and serum total testosterone levels pointed in the same direction, indicating a positive association. In contrast, abnormal morphology and MDA were oriented in the opposite direction, reflecting their negative association with semen quality. The necrozoospermic group clustered closest to MDA, DFI, and abnormal morphology, whereas the fertile control group clustered near progressive motility, vitality, sperm concentration, serum total testosterone, and antioxidant enzyme activities (CAT, SOD, and GPx). Group ellipses illustrate the distribution of samples within each study group.

## 4. Discussion

OS was first identified in 1979 as a key factor in impaired sperm function and is increasingly recognized as a major contributor to male reproductive dysfunction, primarily through the excessive generation of ROS [25]. These molecules trigger lipid peroxidation and induce both nuclear and mitochondrial DNA damage alterations that are not detectable by routine semen analysis [26]. Our study provides an important scientific contribution by simultaneously evaluating the principal OS biomarkers in four pathological groups: oligozoospermic, teratozoospermic, asthenozoospermic, and necrozoospermic men.

A notable strength of this work is the inclusion of the necrozoospermic group, which is rarely investigated in the literature. Furthermore, unlike many previous studies that used normozoospermic men as controls, our control group consisted of fertile men with recently confirmed fertility.

Across all groups, no statistically significant differences were observed in age, abstinence duration, semen volume, or leukocyte counts (*p* > 0.05; Table 1), indicating that these variables do not explain the observed impairments. In contrast, semen quality was significantly altered across the pathological groups, with reduced concentration, motility, vitality, and normal morphology. These results highlight the major role of semen parameters in male fertility and suggest the involvement of noninflammatory factors such as OS [27].

As previously indicated, all infertile groups showed a highly significant increase in the sperm DFI compared with the control group (Table 2). Our data are consistent with the observations reported in the literature [28–30], which found that sperm DNA fragmentation was significantly higher in asthenozoospermic and oligozoospermic patients than in normozoospermic. We also observed significant correlations between semen parameters and DFI; specifically, sperm motility, vitality, and concentration were negatively associated with DNA fragmentation, whereas abnormal morphology showed a positive correlation with higher DFI levels (Table 3). Similar results have been described in previous studies, with negative correlations between DNA damage and motility [31, 32], morphology [33, 34], sperm concentration [32], and vitality [32, 35]. However, a previous study did not corroborate our findings and reported higher sperm DNA fragmentation levels in normozoospermic men compared with groups presenting semen abnormalities [36]. These differences may be attributed to several factors, including the limited DNA repair capacity of spermatozoa, which makes them particularly vulnerable to oxidative DNA damage [9], methodological differences in the assessment of sperm DNA fragmentation, population heterogeneity, and variations in inclusion and exclusion criteria. In our study, we included proven fertile men rather than normozoospermic individuals with unknown fertility status.

Among the mechanisms involved in sperm DNA damage, OS plays a crucial role. In our study, we also observed a positive correlation between DFI and seminal MDA levels, a finding that corroborates previous investigations reporting the same association [28, 32, 37]. Consequently, high DFI disrupts sperm–oocyte interaction and impairs fertilization, which may contribute to assisted reproductive technology (ART) failure. [38]. It is also linked to a higher risk of miscarriage, due to preimplantation or postimplantation losses, and an increased likelihood of congenital anomalies [39].

Regarding OS biomarkers, MDA levels were higher in all infertile groups, with the necrozoospermic group showing the highest values, indicating increased lipid peroxidation. Our results are consistent with previous studies [28, 40], which also reported elevated MDA levels in asthenozoospermic, teratozoospermic, and oligo‐astheno‐teratozoospermic (OAT) patients. Previous investigations have also demonstrated that higher MDA concentrations were negatively correlated with sperm motility [32, 41, 42], morphology [32, 41, 43], and sperm concentration [32, 44, 45]. Moreover, in our study, MDA was negatively correlated with serum total testosterone levels. This observation is supported by evidence from the literature indicating that ROS can disrupt several endocrine axes, including the hypothalamic–pituitary–gonadal (HPG), hypothalamic–pituitary–adrenal (HPA), and hypothalamic–pituitary–thyroid (HPT) axes. Such disruptions contribute to reduced testosterone production, impaired spermatogenesis, and overall male reproductive dysfunction [11, 46].

The activities of the antioxidant enzymes SOD, CAT, and GPx were significantly reduced in all infertile groups compared with the fertile control group, indicating weakened antioxidant defense against ROS. Our findings are consistent with several studies reporting reduced activities of antioxidant enzymes in infertile men [28, 32, 40]. Consistent with previous studies, lower SOD, CAT, and GPx activities were associated with increased OS and reduced sperm motility. Other studies have also shown that decreased antioxidant enzyme activities are associated with lower sperm motility, reduced sperm viability, increased abnormal sperm morphology, and the persistence of residual cytoplasm in pathological groups [32, 47–49].

Among all pathological groups, the necrozoospermic group showed the lowest antioxidant enzyme activities (SOD, CAT, and GPx), the highest MDA concentrations, and the highest DFI values (Table 2). These patterns were clearly reflected in the PCA (Figure 2), where necrozoospermic samples clustered toward MDA, DFI, and abnormal sperm morphology, while being clearly separated from antioxidant biomarkers, highlighting their pronounced oxidative imbalance. These findings may be explained by increased lipid peroxidation leading to the production of reactive aldehydes, such as MDA. These compounds induce nuclear and mitochondrial DNA damage, including the formation of 8‐hydroxy‐2 ^′^‐deoxyguanosine and telomere shortening [50]. Furthermore, ROS disrupt mitochondrial membrane integrity, triggering caspase activation through cytochrome C release and leading to apoptosis [51], which may account for the high proportion of nonviable spermatozoa and the amplification of OS observed in this group.

Overall, our findings demonstrated that a reduced antioxidant profile, combined with elevated seminal MDA concentrations, was closely associated with increased DFI levels and decreased sperm quality. These correlations highlight the essential role of seminal antioxidant enzymes in protecting sperm DNA against OS.

## 5. Limitations

This study has some limitations that should be acknowledged. First, additional hormonal parameters, including luteinizing hormone (LH), follicle‐stimulating hormone (FSH), sex hormone‐binding globulin (SHBG), and free testosterone, were not assessed. Second, direct measurement of ROS was not performed. Third, advanced OS markers, such as oxidation–reduction potential (ORP), were not evaluated.

## 6. Conclusion

This study highlighted the central role of OS in male infertility. Infertile men exhibited significantly elevated seminal MDA levels, reduced antioxidant enzyme activities (SOD, CAT, and GPx), impaired semen parameters, and increased sperm DNA fragmentation. The strong positive correlation between MDA and DFI, together with the negative correlations between MDA and key semen parameters, as well as serum total testosterone levels, highlights the susceptibility of spermatozoa to oxidative damage and endocrine dysfunction. These findings emphasize the crucial contribution of redox imbalance to the deterioration of sperm function. Overall, OS biomarkers may represent promising complementary tools for the assessment of male infertility. However, further studies are needed to recommend their routine use in clinical practice. Future research should focus on establishing standardized protocols for the clinical assessment of OS biomarkers and evaluating strategies aimed at reducing OS and preserving sperm DNA integrity.

## Funding

No funding was received for this manuscript.

## Ethics Statement

The study was approved by the Ethics Committee of the Ibn Rochd University Hospital Center in Casablanca (Ref: 2022/60.CEH.S.KS1.59), in accordance with the Declaration of Helsinki (1975), as revised in 2013. Written informed consent was obtained from all participants prior to their inclusion in the study

## Conflicts of Interest

The authors declare no conflicts of interest.

## Supporting information


**Supporting Information** Additional supporting information can be found online in the Supporting Information section. Supporting information includes a hierarchical clustering heatmap illustrating the distribution and relationships between oxidative stress markers (MDA, CAT, GPx, SOD), sperm DNA fragmentation indices (DFI), semen parameters, and hormonal levels across control and infertile groups.

## Data Availability

The data that support the findings of this study are available from the corresponding author upon reasonable request.
