# Supplementary material for: Evaluation of Oxidative Stress and Its Impact on Sperm DNA Fragmentation and Semen Parameters in Infertile Men
Source: ScientificWorldJournal. 2026 Aug 24;2026:8887567. doi: 10.1155/tswj/8887567 (PMC13501892; doi:10.1155/tswj/8887567)
Supplement: Supplementary file 1 [file TSWJ-2026-8887567-s001.docx]

Supplementary Figure 3: Heatmap cluster showing the distribution of z-score standardized oxidative stress biomarkers, semen quality parameters, DNA fragmentation index, and testosterone levels across study groups. Variables were z-scored and visualized using the pheatmap package.
